# Geographical Association of Bird Species Richness with All-Cause and Cause-Specific Mortality Rates of Local Residents: An Ecological Study in China

**DOI:** 10.3390/life15060875

**Published:** 2025-05-28

**Authors:** Ning Zhang, Jinling You, Qiaochu Xu, Jiacheng Cai, Kelvin P. Jordan, Li Li, Tanchun Yu, Ying Chen

**Affiliations:** 1 Wisdom Lake Academy of Pharmacy, Xi’an Jiaotong-Liverpool University, Suzhou 215123, China; 2 National Center for Chronic and Non-Communicable Disease Control and Prevention, Chinese Center for Disease Control and Prevention, Beijing 100050, China; 3 Department of Geography & Planning, School of Environmental Science, University of Liverpool, Liverpool L69 7ZX, UK; 4 School of Medicine, Keele University, Keele ST5 5BG, UK; 5 Department of Health and Environmental Sciences, School of Sciences, Xi’an Jiaotong-Liverpool University, Suzhou 215123, China; 6 Department of Nutrition and Health Education, National Institute for Nutrition and Health, Chinese Center for Disease Control and Prevention, Beijing 100050, China

**Keywords:** biodiversity, bird species, mortality rate, human health, epidemiology, One Health

## Abstract

The pressing ecological challenges of the twenty-first century underscore the need for biodiversity protection. The “One Health” approach, which integrates human, animal, and environmental health, has become increasingly vital. This study investigates the relationship between bird species richness, an important indicator of biodiversity, and both all-cause and cause-specific mortality rates in China. This nationwide county-level ecological study combined citizen science bird data from the China Bird Report Center, all-cause and cause-specific mortality rates of 2021 from the National Mortality Surveillance System, and county-level statistics of population characteristics, socio-economics, education, and healthcare services. We employed univariate and multivariate linear regressions to explore the association between bird diversity and mortality rates. Overall, data from 421 counties revealed a negative association between bird species richness and all-cause mortality rates, with a regression coefficient (95% confidence interval) of −0.197 (−0.376, −0.017). This study also found significant negative associations between bird species richness and cause-specific mortality rates for several diseases, such as cardiovascular diseases (including cerebrovascular and ischemic heart diseases) and cancers (including lung cancer). The effects of associations were similar between both genders. Our findings underscore the significance of biodiversity conservation for public health and highlight the importance of integrated environmental and health policies.

## 1. Introduction

In the early 21st century, global ecological conservation and biodiversity maintenance have become pressing concerns [1]. Biodiversity is crucial not only for human survival but also as a strategic resource for sustainable socio-economic development [2]. Despite the clear objectives set by the Convention on Biological Diversity, there remains a significant gap between actual conservation efforts and the global consensus, exemplified by the shortfall in protected areas and the ongoing loss of species at an alarming rate [3]. Against this backdrop, “One Health” emphasizes the mutual dependence of environmental, animal, and human health [4]. Its importance is growing as global ecological interactions evolve, with changes in animal populations, such as birds, serving as potential indicators of human disease risk due to their roles as vectors for zoonotic diseases, their sensitivity to environmental changes, and their ability to reflect the health of ecosystems that can influence disease transmission dynamics [5]. Ecosystems that are more stable or resilient are often associated with better human health outcomes [6]. However, the causal relationships between ecosystems and human health are complex, often indirect, and are influenced by various factors over time and space [7]. One of the most extensively studied aspects of this interaction is the influence of species-level biodiversity on human health [8]. Increasing evidence suggests that species diversity is linked to improved psychological and physical health [9,10,11]. For example, a notable correlation was found between self-reported well-being and the estimated plant species richness in urban green spaces [12]. While research on self-reported psychological well-being is growing, studies focusing on well-defined clinical outcomes are less common [13]. The impact of biodiversity on human health, though generally positive, has shown inconsistent results. Different studies report varying relationships between biodiversity and psychological health, with some finding positive associations and others reporting no or even negative effects [14].

China and the USA are part of a select group of 17 “megadiverse” countries, known for their rich indigenous biodiversity and extensive range of species [15]. Although both nations allocate a similar proportion of land to parks, reserves, and protected areas, their strategies for preserving species, populations, and ecosystems differ due to variations in political, legal, economic, and social systems [16]. Law-based approaches established in the USA have outstanding advantages, as seen by the preservation of numerous endangered species [17]. The high expense of species preservation efforts is one of the approach’s flaws, which makes it inappropriate for developing countries. Conversely, agency-based systems like those used in China are complicated. They emphasize the political connections of government agencies [18]. Notably, China has recently increased its attention given to environmental issues in its policy statements and has taken a proactive role in international cooperation to promote the Kunming–Montreal Global Biodiversity Framework (GBF) [19]. A county-level cross-sectional study conducted in the United States found through multivariable regression analysis that areas with higher avian species richness were associated with greater human life expectancy and lower specific disease mortality rates (including cancer and cardiovascular diseases), as well as reduced mortality risk across different age groups [20]. However, to date, no similar studies have been conducted in China. To further investigate whether such a finding is generalizable in a different setting with regard to specific geographical, economic, and cultural factors, we chose to explore these relationships in China.

Birds are widely recognized as effective indicators of biodiversity in regional studies, largely due to their ease of observation and their role as key providers of ecosystem services [21,22]. They are abundant, mobile, and quick to respond to environmental and ecological changes, making their presence or absence a potential signal of environmental shifts. In this study, we utilized bird observation data collected nationwide through citizen science efforts involving non-professional scientists, bird enthusiasts, and volunteers in China. Our objective was to explore the relationship between biodiversity and human health. We hypothesized that bird species richness is associated with all-cause and cause-specific mortality rate data reported in the 2021 National Mortality Surveillance System (NMSS) in China.

## 2. Materials and Methods

### 2.1. Study Design

We conducted a nationwide ecological study to relate biodiversity with human health in China at the county (or county-equivalent) level. In particular, we used two datasets: (1) bird species richness data from the China Bird Report Center (CBRC) as the indicator of biodiversity; and (2) all-cause and cause-specific mortality rate data at the county level from the NMSS as the health outcome variables. In addition, in this study, we enrolled a list of covariates to adjust for potential confounding effects, encompassing population demographics, socio-economics, education, and healthcare service.

### 2.2. Measurement of Biodiversity

Data regarding bird species richness were obtained from CBRC (www.birdreport.cn), a high-quality nationwide online database of bird observations in China [23]. We downloaded a total number of 270,216 bird observation reports on species, families, and locations up to the date of 30 June 2023. These reports were distributed across 34 provinces all over China. A single report may include data for 1 to 120 bird species. Expert reviewers examined each submitted report to filter out questionable entries to guarantee the accuracy of location and species identification. Reviewers were restricted to Chinese birdwatchers who had submitted more than 300 bird species and 100 bird reports [24]. A total number of 2867 county-level data points of bird species richness were sampled in the database, with 1376 bird species and 5,848,819 records. The average number of reports per county was 253. Counties with fewer than 10 reports were excluded from the analysis.

### 2.3. Measurement of Population Health

Population mortality data, including all-cause and cause-specific mortality rates, were obtained from the NMSS, which is administrated by the Chinese Center for Disease Control and Prevention (China’s CDC) [25]. The NMSS is one of the major mortality surveillance systems in China that has estimated all cause-specific mortality rates across all age groups. To ensure provincial representativeness, the NMSS encompassed 605 surveillance points and a population of 323.8 million—representing 24.3% of the total population in China. It was implemented through an iterative process that involved multistage stratification while also considering the sociodemographic characteristics of the population [26]. Given the fluctuation in mortality rate due to the severe pandemic of COVID-19 in China in 2022, we opted to use all-cause and cause-specific mortality data for 2021 to ensure objectiveness while maintaining timeliness. The statistic for the mortality rate was expressed as the number of deaths per 100,000 people. We considered a total of 19 causes of death, including infectious diseases, non-communicable diseases, and injuries, with detailed classifications provided in Table S1.

### 2.4. Covariates

County-level statistics on population demographics (i.e., population density, gender distribution of deaths, and age distribution of deaths), socio-economics (i.e., administrative region land area, gross domestic product per capita, proportions of primary, secondary and tertiary industries, and urbanization rate), length of education, and healthcare service (i.e., number of beds in healthcare institutions per 1000 population) were derived from the 2021 statistical yearbooks of China.

### 2.5. Mapping of Datasets

The databases of bird diversity (*n* = 2867), population mortality rate (*n* = 605), and study covariates (*n* = 2547) were matched by county identity, from which 605 counties were successfully mapped for inclusion. After further removing counties with no more than ten reports on birds (*n* = 184), a total number of 421 counties were finally sampled in this study.

### 2.6. Statistical Analysis

In this study, descriptive statistics were initially presented for all variables of interest, including bird species richness, population demographics, socio-economic factors, education levels, healthcare services, and both all-cause and cause-specific mortality rates. To explore the relationships between these variables, univariate linear regression models were first employed. These models assessed the association between each individual variable and the all-cause mortality rate without adjusting for potential confounding factors. The regression coefficients provided a measure of the impact of a unit change in each exposure variable on the outcome variable, while the R-squared values indicated the overall strength of these associations. Following the univariate analyses, multivariate linear regression models were conducted. These models adjusted for all potential confounding variables to provide a clearer picture of the relationships. For each specific cause of mortality, both univariate and multivariate linear regression analyses were performed to evaluate the association with bird species richness. Additionally, a subgroup analysis stratified by gender was conducted. This allowed for the examination of whether the associations between bird species richness and mortality rates differed between males and females, providing further insight into the potential health benefits of biodiversity across different demographic groups.

Linear regression models were estimated using ordinary least squares (OLS) with a robust standard error to reduce the effect of heteroscedasticity. Where cause-specific mortality rates were particularly low for certain conditions—reported as 0 in significant proportions of the studied counties—we repeated analyses with the Tobit regression models as a sensitivity analysis to ensure the robustness of the results, given that the OLS linear regression is susceptible to biased estimates. All analyses were performed with STATA software (version 16.0). A two-tailed *p*-value of less than 0.05 was deemed statistically significant in all analyses.

## 3. Results

Table 1 presents the county-level descriptive statistics for 421 counties, covering population demographics, socio-economic factors, education, and healthcare services. The data highlight the diverse economic structures and uneven development across these counties. This diversity is evident in the varied industry percentages and urbanization rates, with some counties being more industrialized and others relying more heavily on agriculture or service sectors. In terms of healthcare resources, the median number of beds available in healthcare institutions per 1000 population was 5.6, indicating the capacity for medical care across different regions. Education levels also varied, with a median length of education recorded at 9.1 years.

**Figure 1:**
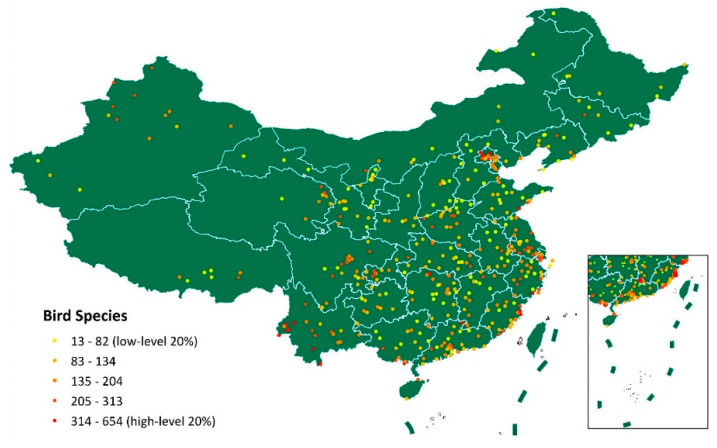
Geographical distribution of the 421 studied counties with information on bird species richness.

**Figure 2:**
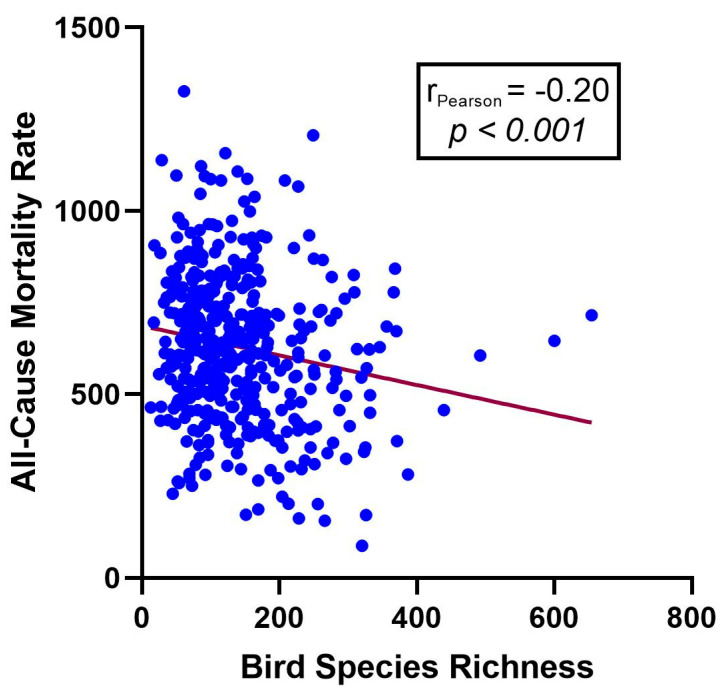
Scatter plot with a linear fitted line of number of bird species with rate of all-cause mortality per 100,000 population.

The geographical distribution of studied counties and the variety of bird species richness in these counties are demonstrated in Figure 1. On average, 140.72 bird species were observed per county, with a standard deviation of 87.37. Higher bird species richness was particularly notable in the coastal and western regions, such as Yunnan Province. The average all-cause mortality rate across these counties was 545.54 per 100,000 people, with a standard deviation of 189.35. The initial correlation analysis indicated a negative relationship between bird species richness and all-cause mortality rates (Pearson’s correlation coefficient: −0.2, with a *p*-value less than 0.001), as shown in Figure 2. Descriptive statistics on the cause-specific mortality rates in the studied counties are shown in Table S1. The top three major causes of death were cardiovascular diseases (mean (SD), 247.79 (115.93), per 100,000 populations), cancers (140.1 (61.26)), and respiratory diseases (45.87 (31.83)).

In the univariate regression analyses, most exposure variables showed a significant association with the all-cause mortality rate (see “separate univariate models”, Table 2). The urbanization rate accounted for 17.4% of the variance in all-cause mortality rates, followed by per capita GDP at 15.2%. Bird species richness, the sole biodiversity indicator, explained 3.3% of the variance. In the multivariate regression model, we found that each additional bird species was significantly linked to a 0.197 decrease in the all-cause mortality rate after adjusting for other exposure variables (see ”multivariate model”, Table 2).

The results for the relationships between the richness of bird species and rates of cause-specific mortality are summarized in Table 3. From a broad classification perspective, out of 19 causes of death, 5 causes showed a significant association with bird species richness. After adjustment for confounding factors, negative associations were observed with cardiovascular diseases (regression coefficient (95% confidence interval (CI), −0.211 (−0.315, −0.106)) and cancers (−0.057 (−0.113, −0.001)), both demonstrating considerable effect sizes (Cohen’s f^2^ = 0.558 and 0.325, respectively). Significant associations were specifically noted for mortality from cerebrovascular disease, ischemic heart disease, and lung cancer. Although associations with neurological and mental disorders (0.0079 (0.0007, 0.0151)) and musculoskeletal and connective tissue disorders (0.0028 (0.0007, 0.0048)) were identified, the effect sizes and associated mortality rates were notably smaller.

The subgroup analyses conducted in this study revealed consistent patterns across both male and female populations. Specifically, there were notable negative associations between bird species richness and mortality rates for a wide range of diseases. This suggests that areas with higher bird species diversity tend to have lower mortality rates from these diseases, regardless of gender. This consistency across genders strengthens the evidence for the role of biodiversity as a beneficial factor in public health outcomes (Table 4).

Sensitivity analysis, which repeated several analyses with the Tobit regression modeling where the outcome variable was characterized by lots of zeros, resulted in similar findings.

## 4. Discussion

To the best of our knowledge, this is the first study to explore the relationship between bird species richness with important indicators of population health at the national scale in China. In this study, we found that a reduced rate of all-cause mortality was significantly associated with increased bird species richness. Birds have strong mobility and respond quickly to environmental and ecological changes, making them a reliable indicator of biodiversity [22]. After adjusting for other confounding factors, this association was stronger for some causes of death with high disease burdens, where increases in bird species per unit affected mortality to a greater extent, e.g., cardiovascular diseases (e.g., ischemic heart disease and cerebrovascular disease), and cancers (e.g., lung cancer). The results in this study showed high consistency with our previous studies conducted in the USA [20,27]. From an overall perspective, a higher species richness of birds was significantly associated with a longer average human life expectancy and reduced mortality rates from overall neoplasm and cardiovascular diseases [20]. In terms of specific disease mortalities, bird species richness was significantly associated with three of the five most common cancers, including lung cancer, breast cancer, and colon and rectal cancer, as well as with cerebrovascular disease and ischemic heart disease among cardiovascular diseases [27]. Such results are consistent across two regions that differ in terms of economic development, cultural identity, politics, and geographic characteristics.

Our findings indicate that biodiversity has the potential to lower the risk of various cause-specific mortality, reaching agreement on the “One Health” approach. ”One Health” recognizes that human health is closely connected to the health of animals, our shared environment, and transdisciplinary efforts [28]. In the current study, analyses of cause-specific mortality rates revealed that bird species richness had the most significant association with cardiovascular-disease-related mortality, followed by malignant-tumor mortality. These conditions are regarded as the most concerning burden of human health problems. Specifically, over 80% of deaths from cardiovascular diseases can be attributed to ischemic heart disease and cerebrovascular disease [29]. The findings also emphasize the potential threats underlying the synergistic relationship between biodiversity and human health. One possible mechanism is that areas with a greater variety of bird species may also have a higher quality environment in general, which may have impacts on the linkage between biodiversity and mortalities of cardiovascular diseases and malignant tumors. In addition to air pollution and environmental chemicals, other similar threats were proposed in the recent literature, including nighttime light exposure, biotoxins, and nutritional deficiencies [30,31,32,33,34,35]. For sure, our methodology is not able to provide strong causal inferences, however, the study design is indeed a practical and feasible approach. There is also a fundamental principal framework signifying that the association between biodiversity and human health is meaningful [8]. Some potential mechanisms that establish the pathways between biodiversity and human health can demonstrate this synergistic interaction, such as the reduction in air contamination. Cancer is believed to result from a mix of environmental and genetic factors [36]. External influences like air pollution significantly contribute to various human diseases such as respiratory diseases, cardiovascular diseases, and lung cancer [36]. Certain environmental chemicals and air pollutants act as carcinogens, targeting specific biological pathways or increasing susceptibility to carcinogenesis [37]. Notably, particulate matter (PM) in air pollution can cause inflammatory changes in lung tissue, promoting cancer cell growth, and potentially increasing the incidence of ischemic heart diseases and death from cerebrovascular disease [38,39]. At the other end of the pathway, climate change and long-term exposure to air pollution are proven to affect human health and cause a loss of biodiversity [40]. The sensitivity of birds—as a representative of biodiversity—to environmental change often reflects the overall environmental quality of a given area [41]. The mechanisms by which biodiversity interacts with human health are likely to be multiple, and more known pathways need to be further investigated to reveal the common drivers behind the observed associations between biodiversity and health.

However, biodiversity might also have its own direct functional effects in enhancing human health. Research using Germany as a case study examined the relationship between species diversity and human health at the regional scale [42]. The study used species richness and the abundance of birds and plants as measures of species diversity, alongside self-reported mental health and physical health scales. Significant positive associations were found between plant and bird species richness and mental health. Another study conducted in Michigan, USA, identified a significant association between the increase in bird species diversity and a reduction in hospitalizations due to anxiety or mood disorders [43]. These suggested that biodiversity could contribute positively to the improvement of severe mental health conditions. In addition to bird or plant species richness, another common biodiversity indicator, greenness (or green spaces), is closely linked to both physical and mental health [12,44,45]. A systematic review of the association between residential green space exposure and adult mortality found that most of the 12 studies conducted in North America, Europe, and Oceania showed a reduced risk of cardiovascular disease deaths in areas with higher levels of residential green space [46]. However, green spaces are more likely to be associated with socio-economic factors. In areas or communities with better economic development, there may be a higher coverage of green spaces. In contrast, the relationship between birds and humans is more independent, particularly at regional levels. In general, there is growing evidence of associations between biodiversity and human health and mortality from specific diseases, whether directly or through co-driven synergistic associations.

Overall, this is the first large nationwide study in China to discuss the association between biodiversity and human health. It collected data on demographics, socio-economic conditions, education, and healthcare services at the county level, as recorded by statistical yearbooks of China. This allowed for detailed adjustments of confounding variables in the statistical analyses. Our findings provided an ecological association between bird diversity and mortalities of certain conditions at the smallest administrative units in the board region. We assessed the association between bird diversity and mortality rate, encompassing analyses across all-cause mortality and a spectrum of detailed causes of death. We analyzed the mortality rates of a total of 19 causes of death, including cancer and cardiovascular diseases, infectious diseases, chronic non-communicable diseases, and injuries, as well as 36 more specific causes. Nevertheless, there are several limitations in this study. The inherent ecological fallacy in ecological studies cannot make conclusions on an individual level. Typical issues associated with ecological studies, such as bias due to migration, could also influence the results. Additionally, our statistical analyses were developed from cross-sectional studies, whereas in reality there are long-term interactions between environmental exposures and health responses. Meanwhile, bird observations might be affected by potential human activities, such as organized bird watching. China’s vast geographical expanse resulted in a clear gradient of biodiversity, where the number of bird species tended to increase from north to south and from west to east. Consequently, it is important to consider these regional variations when interpreting the relationship between bird species richness and human health outcomes. Another limitation of this study was the assumption that the number of bird species recorded was complete. In reality, these numbers might only represent a fraction of the species that actually occur in a given county. In many areas, particularly those with lower reporting rates, the true number of species could be underestimated, while urban areas might have more complete records due to higher birdwatching activity. This potential bias should be considered when interpreting the association between bird species richness and human disease incidence.

## 5. Conclusions

In conclusion, this wide-ranging ecological study is among the first to report a significant inverse association between bird species richness and the all-cause mortality rate, with a distinct emphasis on mortality attributed to cardiovascular ailments and malignant tumors. The results indicate that regions with greater bird species richness demonstrate significantly lower mortality rates after adjustments for other confounding factors. Although our approach cannot reveal causality, our findings lend epidemiological support to existing frameworks that underscore the inherent value of biodiversity in shaping human health outcomes. For more direct proof and the mechanisms, future research investigating the relationship between various biodiversity indicators and health outcomes is necessary.

## Figures and Tables

**Table 1:** Summary descriptive statistics for 421 studied Chinese counties in 2021.

Variable	Number (%) or Median (IQR)	Mean (SD)
Biodiversity		
Bird species richness	119.50 (79.00, 177.50)	140.72 (87.37)
Human health		
All-cause mortality rate, per 100,000 population	628.54 (483.34, 767.24)	632.93 (215.32)
Population demographics		
Population density, person/${km}^{2}$	417.59 (162.46, 979.41)	1702.06 (4175.29)
Male distribution of deaths, %	57.43% (56.30%, 57.81%)	57.61 (1.99)
Age distribution of deaths, %		
0–9 years	0.44% (0.27%, 0.73%)	0.63% (0.76%)
10–19 years	0.33% (0.22%, 0.51%)	0.42% (0.35%)
20–29 years	0.53% (0.36%, 0.79%)	0.65% (0.92%)
30–39 years	1.43% (1.06%, 1.92%)	1.64% (0.92%)
40–49 years	3.57% (2.82%, 4.53%)	3.92% (1.69%)
50–59 years	10.04% (8.72%, 11.58%)	10.27% (2.42%)
60–69 years	15.86% (14.21%, 17.95%)	16.31% (3.19%)
70–79 years	25.32% (23.06%, 28.10%)	25.58% (3.59%)
80 years and over	41.20% (35.65%, 45.89%)	40.59% (8.26%)
Socio-economics		
Administrative region land area, ${km}^{2}$	1523 (664, 2673)	2877.00 (6925.01)
Per capita GDP, CNY	71,435 (40,326.56, 108,067.10)	88,415.67 (74,554)
Primary industry, %	10.11% (3.52%, 18.51%)	12.16% (10.41%)
Secondary industry, %	36.85% (26.49%, 46.70%)	36.75% (14.58%)
Tertiary industry, %	48.53% (42.04%, 59.08%)	50.53% (15.04%)
Urbanization rate, %	60.27% (47.17%, 83.04%)	64.74% (22.15%)
Education and healthcare service		
Number of beds, per 1000 population	5.57 (4.6, 7.27)	6.23 (2.98)
Length of education, years	9.19 (8.63, 10.45)	9.51 (1.26)

IQR, interquartile range; SD, standard deviation; GDP, gross domestic product; CNY, Chinese Yuan.

**Table 2:** Regression models on the relationship between studied exposure variables and rate of all-cause mortality.

	All-Cause Mortality Rate, per 100,000 Population (Outcome Variable)					
Exposure Variable	Separate Univariate Models			Multivariate Model		
	Regression Coefficient (95% Confidence Interval)	*p*-Value	R^2^	Regression Coefficient (95% Confidence Interval)	*p*-Value	R^2^
Biodiversity						
Bird species richness	−0.4462 (−0.6977, −0.1947)	0.001	0.033	−0.1967 (−0.3763, −0.0173)	0.032	0.356
Population characteristics						
Age ≥ 65 (%)	1.8689 (−1.5017, 5.2394)	0.276	0.003	7.3490 (3.6895, 11.0085)	<0.001	
Male (%)	−13.8474 (−24.1776, −3.5173)	0.009	0.017	−9.3523 (−18.4885, −0.2161)	0.045	
Population density (per km^2^)	−0.0028 (−0.0078, 0.0021)	0.263	0.003	0.0113 (0.0060, 0.0167)	<0.001	
Socio-economics						
Per capita GDP (CNY)	−0.0011 (−0.0013, −0.0008)	<0.001	0.152	−0.0007 (−0.0010, −0.0004)	<0.001	
Primary industry (%)	7.4521 (5.8875, 9.3168)	<0.001	0.130	4.6799 (1.9361, 7.4236)	0.001	
Secondary industry (%)	−0.1424 (−1.5685, 1.2846)	0.845	<0.001	2.0686 (0.6538, 3.4833)	0.004	
Tertiary industry (%)	−2.9600 (−4.3166, −1.6095)	<0.001	0.043	−0.4589 (−1.8335, 0.9156)	0.512	
Urbanization rate (%)	−4.0583 (−4.9118, −3.2047)	<0.001	0.174	−3.0276 (−5.3016, −0.7537)	0.009	
Education and healthcare service						
Number of beds (per 1000 persons)	−10.1644 (−17.0946, −3.2342)	0.004	0.020	3.5006 (−2.3015, 9.3027)	0.236	
Length of education (years)	−62.7881 (−78.1801, −47.3960)	<0.001	0.134	−11.3529 (−40.6368, 17.9310)	0.446	

GDP, gross domestic product; CNY, Chinese Yuan; R^2^, demonstrating the proportion of variance in the mortality rate explained by the exposure variable(s).

**Table 3:** Relationships between richness of bird species and rates of cause-specific mortality.

Cause of Death		Bird Species Richness (Exposure Variable)				
		Univariate Analyses (Unadjusted)			Multivariate Analyses (Adjusted)	
		Regression Coefficient (95% Confidence Interval)	$R^{2}$	*p*-Value	Regression Coefficient (95% Confidence Interval)	*p*-Value
Infectious diseases, maternal and infant diseases, and nutritional deficiencies						
	Infectious diseases	−0.0051 (−0.01203, 0.0017)	0.007	0.142	−0.0030 (−0.0093, 0.0032)	0.340
	HIV/AIDS, sexually transmitted diseases, and tuberculosis	−0.0016 (−0.0062, 0.0030)	0.002	0.487	−0.0008 (−0.0050, 0.0037)	0.716
	Hepatitis	−0.0043 (−0.0071, −0.0015)	0.020	0.003	−0.0030 (−0.0058, −0.0003)	0.028
	Respiratory infection	−0.0002 (−0.0035, 0.0031)	<0.001	0.905	0.0002 (−0.0032, 0.0036)	0.908
	Nutritional deficiencies	0.0041 (−0.0022, 0.0104)	0.007	0.199	0.0051 (−0.0018, 0.0120)	0.150
	Parasitic diseases	−0.0003 (−0.0010, 0.0004)	0.002	0.382	−0.0003 (−0.0010, 0.0004)	0.398
	Pregnancy, childbirth, and postnatal complications	−0.0001 (−0.0002, 0.0000)	0.004	0.146	−0.0001 (−0.0002, 0.0006)	0.309
Chronic non-communicable diseases						
	Cardiovascular diseases	−0.3112 (−0.4543, −0.1682)	0.055	<0.001	−0.2107 (−0.3152, −0.1062)	<0.001
	Cerebrovascular disease	−0.1410 (−0.2161, −0.0659)	0.043	<0.001	−0.0871 (−0.1398, −0.0344)	0.001
	Ischemic heart disease	−0.1705 (−0.2366, −0.1044)	0.060	<0.001	−0.1395 (−0.1973, −0.0816)	<0.001
	Hypertension	0.0046 (−0.0100, 0.0192)	0.001	0.535	0.0132 (−0.0009, 0.0273)	0.067
	Rheumatic heart disease	−0.0014 (−0.0054, 0.0026)	0.001	0.492	0.0014 (−0.0027, 0.0055)	0.501
	Cardiomyopathy	−0.0004 (−0.0000, 0.0008)	0.005	0.051	0.0004 (−0.0000, 0.0009)	0.054
	Cancers	−0.1031 (−0.1628, −0.0434)	0.022	0.001	−0.0573 (−0.1134, −0.0012)	0.045
	Lung cancer	−0.0352 (−0.0545, −0.0158)	0.021	<0.001	−0.0215 (−0.0415, −0.0014)	0.036
	Colorectal cancer	−0.0008 (−0.0070, 0.0053)	0.0001	0.788	0.0019 (−0.0038, 0.0077)	0.516
	Lymphoma and multiple myeloma	0.0008 (−0.0013, 0.0029)	0.001	0.437	0.0013 (−0.0006, 0.0032)	0.174
	Leukemia	−0.0020 (−0.0035, −0.0005)	0.012	0.011	−0.0012 (−0.0026, 0.0001)	0.084
	Prostate cancer	−0.0006 (−0.0019, 0.0007)	0.002	0.371	−0.0003 (−0.0016, 0.0010)	0.635
	Ovarian cancer	−0.0004 (−0.0014, 0.0006)	0.003	0.432	−0.0003 (−0.0012, 0.0007)	0.592
	Stomach cancer	−0.0031 (−0.0065, 0.0003)	0.004	0.071	−0.0033 (−0.0067, 0.0002)	0.064
	Endometrial cancer	−0.0008 (−0.0016, 0.0001)	0.008	0.065	−0.0004 (−0.0011, 0.0004)	0.318
	Liver cancer	−0.0025 (−0.0047, −0.0003)	0.008	0.028	−0.0018 (−0.0039, 0.0004)	0.104
	Mammary cancer	−0.0002 (−0.0005, 0.0011)	0.001	0.570	0.0006 (−0.0001, 0.0014)	0.107
	Esophageal cancer	−0.0001 (−0.0015, 0.0013)	0.001	0.907	0.0003 (−0.0013, 0.0019)	0.711
	Skin cancer	−0.0003 (−0.0008, 0.0003)	0.001	0.353	0.0001 (−0.0005, 0.0007)	0.785
	Pancreatic cancer	−0.0084 (−0.0196, 0.0027)	0.006	0.137	−0.0011 (−0.0119, 0.0097)	0.837
	Cervical cancer	−0.0008 (−0.0015, −0.0001)	0.0115	0.023	−0.0006 (−0.0012, −0.0001)	0.084
	Bladder cancer	−0.0007 (−0.0014, 0.0001)	0.010	0.068	−0.0007 (−0.0015, 0.0001)	0.090
	Respiratory diseases	−0.0034 (−0.0375, 0.0305)	0.0001	0.842	0.0197 (−0.0125, 0.0518)	0.230
	Chronic obstructive pulmonary disease	−0.0070 (−0.0383, 0.0243)	0.0004	0.660	0.0147 (−0.0149, 0.0443)	0.331
	Asthma	0.0015 (−0.0002, 0.0032)	0.008	0.088	0.0019 (0.0001, 0.0037)	0.038
	Endocrine nutritional metabolic diseases	−0.0084 (−0.0196, 0.0027)	0.006	0.137	−0.0011 (−0.0119, 0.0097)	0.837
	Diabetes	−0.0127 (−0.0211, −0.0045)	0.018	0.001	−0.0065 (−0.0145, 0.0015)	0.109
	Digestive disorders	0.0008 (−0.0087, 0.0102)	0.001	0.872	0.0078 (−0.0005, 0.0161)	0.067
	Neurological and mental disorders	0.0056 (0.0011, 0.0122)	0.005	0.025	0.0079 (0.0007, 0.0151)	0.030
	Dementia	0.0031 (−0.0016, 0.0077)	0.003	0.196	0.0040 (−0.0009, 0.0089)	0.112
	Epilepsy	0.0001 (−0.0009, 0.0009)	<0.001	0.953	0.0003 (−0.0005, 0.0011)	0.426
	Schizophrenic	0.0003 (−0.0006, 0.0010)	0.003	0.332	0.0006 (−0.0001, 0.0012)	0.065
	Unipolar mental depression	−0.0002 (−0.0003, 0.0001)	0.005	0.069	−0.0002 (−0.0003, 0.0001)	0.072
	Bipolar disorder	0.0001 (−0.0000, 0.0001)	0.001	0.495	0.0001 (−0.0005, 0.0001)	0.436
	Urogenital diseases	−0.0004 (−0.0057, 0.0048)	0.0001	0.867	0.0038 (−0.0002, 0.0077)	0.065
	Musculoskeletal and connective tissue disorders	0.0018 (−0.0003, 0.0039)	0.006	0.100	0.0028 (0.0007, 0.0048)	0.010
	Hematopoietic diseases	−0.0001 (−0.0015, 0.0013)	0.0001	0.907	0.0003 (−0.0013, 0.0019)	0.711
	Congenital anomalies	0.0002 (−0.0006, 0.0011)	0.001	0.570	0.0006 (−0.0001, 0.0014)	0.107
	Sensory diseases	0.0001 (−0.0001, 0.0001)	0.001	0.625	−0.0001 (−0.0000, 0.0001)	0.318
	Oral diseases	−0.0001 (−0.0001, 0.0000)	0.005	0.146	−0.0001 (−0.0001, 0.0000)	0.192
Injuries						
	Accidents	0.0026 (−0.0154, 0.0207)	0.0002	0.775	0.0181 (0.0026, 0.0335)	0.022
	Traffic accidents	−0.0087 (−0.0158, −0.0017)	0.014	0.016	−0.0052 (−0.0114,0.0011)	0.107
	Accidental falls	0.0071 (−0.0009, 0.0152)	0.005	0.082	0.0137 (0.0059,0.0216)	0.001
	Accidental poisoning	0.0008 (−0.0027, 0.0042)	0.001	0.665	0.0024 (−0.0005,0.0054)	0.108
	Fire	−0.00001 (−0.0007,0.0006)	0.000	0.961	0.0001 (−0.0005,0.0008)	0.684
	Unintentional injuries	0.0012 (−0.0038, 0.0063)	0.004	0.632	0.0039 (−0.0011, 0.0089)	0.122
	Suicide and sequelae	0.0013 (−0.0037, 0.0062)	0.001	0.609	0.0036 (−0.0011, 0.0088)	0.123
	Homicide and sequelae	−0.0001 (−0.0004, 0.0003)	0.0002	0.754	0.0001 (−0.0003, 0.0004)	0.796

Multivariate analyses adjusted for all the studied covariates; R^2^, demonstrating the proportion of variance in the mortality rate explained by bird species richness.

**Table 4:** Relationships between richness of bird species and rates of cause-specific mortality by gender.

Cause of Death		Bird Species Richness (Exposure Variable)			
		Adjusted Analyses in Females		Adjusted Analyses in Males	
		Regression Coefficient (95% Confidence Interval)	*p*-Value	Regression Coefficient (95% Confidence Interval)	*p*-Value
Infectious diseases, maternal and infant diseases, and nutritional deficiencies					
	Infectious diseases	−0.0017 (−0.0066, 0.0032)	0.493	−0.0063 (−0.0167, 0.0040)	0.229
	HIV/AIDS, STDs, and tuberculosis	−0.0011 (−0.0043, 0.0021)	0.496	−0.0018 (−0.0086, 0.0049)	0.595
	Hepatitis	−0.0018 (−0.0416, −0.0005)	0.129	−0.0052 (−0.0094, −0.0010)	0.016
	Respiratory infection	−0.0004 (−0.0038, 0.0029)	0.792	0.0001 (−0.0043, 0.0044)	0.982
	Nutritional deficiencies	0.0052 (−0.0025, 0.0134)	0.182	0.0043 (−0.0018, 0.0105)	0.167
	Parasitic diseases	−0.0004 (−0.0012, 0.0005)	0.385	−0.0003 (−0.0011, 0.0004)	0.389
	Pregnancy, childbirth, and postnatal complications	−0.0002 (−0.0004, 0.0001)	0.239	-	-
Chronic non-communicable diseases					
	Cardiovascular diseases	−0.2048 (−0.3068, −0.1027)	<0.001	−0.2756 (−0.4037, −0.1475)	<0.001
	Cerebrovascular disease	−0.0818 (−0.1308, −0.0328)	0.001	−0.1207 (−0.1866, −0.0548)	<0.001
	Ischemic heart disease	−0.1354 (−0.1935, −0.0774)	<0.001	−0.1698 (−0.2391, −0.1006)	<0.001
	Hypertension	0.0139 (−0.0033, 0.0310)	0.241	0.0122 (−0.0019, 0.0264)	0.090
	Rheumatic heart disease	0.0021 (−0.0026, 0.0069)	0.379	0.0010 (−0.0330, 0.0053)	0.653
	Cardiomyopathy	0.0002 (−0.0006, 0.0010)	0.591	0.0006 (−0.0000, 0.0012)	0.065
	Cancers	−0.0518 (−0.0993, −0.0043)	0.033	−0.0865 (−0.1630, −0.0107)	0.027
	Lung cancer	−0.0172 (−0.0325, −0.0020)	0.027	−0.0328 (−0.0621, −0.0035)	0.028
	Colorectal cancer	−0.0009 (−0.0067, 0.0048)	0.748	0.0033 (−0.0046, 0.0113)	0.406
	Lymphoma and multiple myeloma	0.0017 (−0.0002, 0.0036)	0.075	0.0008 (−0.0017, 0.0033)	0.536
	Leukemia	−0.0010 (−0.0026, 0.0007)	0.243	−0.0020 (−0.0039, −0.0001)	0.043
	Prostate cancer	-	-	−0.0010 (−0.0035, 0.0016)	0.462
	Ovarian cancer	−0.0011 (−0.0032, 0.0012)	0.371	-	-
	Stomach cancer	−0.0024 (−0.0047, −0.0001)	0.045	−0.0049 (−0.0100, 0.0006)	0.061
	Endometrial cancer	−0.0009 (−0.0027, 0.0008)	0.303	-	-
	Liver cancer	−0.0013 (−0.0027, −0.0001)	0.074	−0.0027 (−0.0059, 0.0004)	0.090
	Mammary cancer	0.0005 (−0.0006, 0.0015)	0.385	0.0006 (−0.0004, 0.0017)	0.248
	Esophageal cancer	−0.0003 (−0.0022, 0.0015)	0.724	0.0005 (−0.0014, 0.0024)	0.606
	Skin cancer	0.0002 (−0.0010, 0.0015)	0.712	0.0003 (−0.0009, 0.0015)	0.630
	Pancreatic cancer	−0.0008 (−0.0130, 0.0113)	0.890	−0.0054 (−0.0167, 0.0059)	0.346
	Cervical cancer	−0.0013 (−0.0027, −0.0001)	0.045	-	-
	Bladder cancer	−0.0003 (−0.0007, 0.0000)	0.079	−0.0012 (−0.0027, 0.0025)	0.105
	Respiratory diseases	0.0080 (−0.0219, 0.0379)	0.599	0.0243 (−0.0148, 0.0635)	0.223
	Chronic obstructive pulmonary disease	0.0080 (−0.0193, 0.0353)	0.567	0.0192 (−0.0164, 0.0548)	0.289
	Asthma	0.0010 (−0.0006, 0.0028)	0.216	0.0027 (0.0004, 0.0050)	0.021
	Endocrine nutritional metabolic diseases	−0.0009 (−0.0130, 0.0113)	0.890	−0.0054 (−0.0167, 0.0059)	0.346
	Diabetes	−0.0068 (−0.0160, 0.0024)	0.149	−0.0099 (−0.0187, −0.0011)	0.027
	Digestive disorders	0.0005 (−0.0072, 0.0083)	0.895	0.0120 (0.0001, 0.0240)	0.048
	Neurological and mental disorders	0.0100 (0.0014, 0.0187)	0.023	0.0070 (−0.0001, 0.0142)	0.054
	Dementia	0.0054 (−0.0008, 0.0117)	0.088	0.0025 (−0.0019, 0.0069)	0.261
	Epilepsy	0.0010 (−0.0011, 0.0032)	0.345	0.0001 (−0.0012, 0.0013)	0.910
	Schizophrenic	0.0004 (−0.0004, 0.0013)	0.320	0.0006 (−0.0001, 0.0013)	0.096
	Unipolar mental depression	−0.0002 (−0.0005, −0.001)	0.263	−0.0002 (−0.0004, 0.0001)	0.069
	Bipolar disorder	0.0001 (−00001, 0.0001)	0.820	0.0001 (−0.0001, 0.0002)	0.425
	Urogenital diseases	0.0019 (−0.0026, 0.0063)	0.407	0.0054 (0.0003, 0.0105)	0.036
	Musculoskeletal and connective tissue disorders	0.0028 (0.0003, 0.0053)	0.027	0.0023 (0.0001, 0.0045)	0.038
	Hematopoietic diseases	−0.0003 (−0.0022, 0.0015)	0.724	0.0005 (−0.0014, 0.0024)	0.606
	Congenital anomalies	0.0005 (−0.0006, 0.0015)	0.385	0.0006 (−0.0004, 0.0017)	0.248
	Sensory diseases	−0.0003 (−0.0001, 0.0000)	0.296	0.0002 (−0.0001, 0.0029)	0.180
	Oral diseases	0.0001 (−0.0001, 0.0001)	0.240	−0.0002 (−0.0003, −0.0001)	0.037
Injuries					
	Accidents	0.0138 (0.0014, 0.0262)	0.029	0.0194 (−0.0018, 0.0410)	0.072
	Traffic accidents	−0.0029 (−0.0070, 0.0011)	0.153	−0.0095 (−0.0189, −0.0001)	0.048
	Accidental falls	0.0116 (0.0043, 0.0189)	0.002	0.0161 (0.0059, 0.0262)	0.002
	Accidental poisoning	0.0005 (−0.0012, 0.0021)	0.568	0.0042 (−0.0009, 0.0093)	0.106
	Fire	0.0014 (−0.0005,0.0008)	0.664	0.0019 (−0.0010,0.0014)	0.756
	Unintentional injuries	0.0030 (−0.0023, 0.0084)	0.265	0.0039 (−0.0021, 0.0098)	0.202
	Suicide and sequelae	0.0029 (−0.0024, 0.0008)	0.287	0.0029 (−0.0029, 0.0087)	0.325
	Homicide and sequelae	−0.0003 (−0.0011, 0.0005)	0.490	0.0002 (−0.0007, 0.0011)	0.612

Analyses adjusted for all the studied covariates.

## Data Availability

The data that support the findings of this study are available from the Chinese Center for Disease Control and Prevention but restrictions apply to the availability of these data, which were used under license for the current study, and so are not publicly available. Data are however available from the authors upon reasonable request and with permission of the Chinese Center for Disease Control and Prevention.

## Supplementary Materials

The following supporting information can be downloaded at https://www.mdpi.com/article/10.3390/life15060875/s1, Table S1: County-level statistics on cause-specific mortality rates in 421 studied Chinese counties in 2021.
