# Supplementary material for: Geographical Association of Bird Species Richness with All-Cause and Cause-Specific Mortality Rates of Local Residents: An Ecological Study in China
Source: Life (Basel). 2025 May 28;15(6):875. doi: 10.3390/life15060875 (PMC12193953; doi:10.3390/life15060875)
Supplement: Supplementary file 1 [file life-15-00875-s001.zip › life-3613676-supplementary.pdf]

Table S1. County-level statistics on cause-specific mortality rates on 421 studied Chinese counties in 2021.

Cause of death	Number of deaths per 100,000 population				
	Median	25th Percentile	75th Percentile	Mean	Standard deviation
Infectious diseases, maternal and infant disease, and nutritional deficiencies					
Infectious diseases	4.90	3.05	7.75	6.04	5.57
Hepatitis	1.83	0.81	3.22	2.49	2.66
HIV, tuberculosis, and sexually transmitted disease	1.50	0.81	2.82	2.31	3.43
Respiratory infection	1.41	0.55	2.95	2.53	3.42
Nutritional deficiencies	0.54	0.14	1.43	1.67	4.28
Parasitic diseases	0.00	0.00	0.00	0.09	0.68
Complications during pregnancy, childbirth and the postnatal period	0.00	0.00	0.00	0.04	0.13
Chronic non-communicable diseases					
Cardiovascular diseases	241.97	163.01	311.16	247.79	115.93
Cerebrovascular disease	113.66	76.92	152.92	118.68	59.53
Ischemic heart disease	80.06	51.21	119.74	93.41	61.00
Hypertension	14.94	8.34	25.73	19.42	16.74
Rheumatic heart disease	1.62	0.86	3.65	3.22	4.55
Cardiomyopathy	0.20	0.00	0.43	0.33	0.50
Cancers	127.43	97.75	174.65	140.10	61.26
Lung cancer	35.81	24.03	51.43	39.20	21.77
Pancreatic cancer	16.51	11.36	23.13	18.02	9.63

Colorectal cancer	10.58	7.17	14.89	11.46	6.05
Leukemia	2.91	2.15	4.02	3.16	1.55
Lymphoma and multiple myeloma	2.50	1.45	4.00	2.86	1.94
Prostate cancer (only in males)	1.66	1.04	2.55	1.88	1.30
Ovarian cancer (only in females)	1.20	0.68	1.81	1.32	0.91
Esophageal cancer	0.94	0.60	1.45	1.16	1.00
Endometrial cancer (only in females)	0.91	0.54	1.40	1.02	0.74
Mammary cancer	0.81	0.46	1.32	0.96	0.75
Skin cancer	0.54	0.27	0.97	0.68	0.61
Liver cancer	0.16	0.00	1.08	1.10	2.36
Stomach cancer	0.10	0.00	1.20	1.61	4.33
Bladder cancer	0.00	0.00	0.40	0.32	0.61
Cervical cancer (only in females)	0.00	0.00	0.30	0.31	0.64
Respiratory diseases	37.00	24.46	56.33	45.87	31.83
Chronic obstructive pulmonary disease	24.11	14.10	43.09	34.07	30.62
Asthma	0.67	0.32	1.43	1.16	1.48
Endocrine nutritional metabolic diseases	16.51	11.36	23.13	18.02	9.63
Diabetes	15.03	10.17	20.25	15.85	8.38
Digestive disorders	11.91	8.00	17.43	14.26	9.29
Neurological and mental disorders	7.54	4.62	12.16	9.31	6.92
Dementia	2.35	0.91	5.11	3.96	4.90
Epilepsy	0.64	0.31	1.02	0.79	0.88
Schizophrenic	0.23	0.00	0.55	0.41	0.53
Unipolar mental depression	0.00	0.00	0.18	0.11	0.19
Bipolar disorder	0.00	0.00	0.00	0.02	0.08
Urogenital diseases	5.88	3.63	8.25	6.59	3.94

Musculoskeletal and connective tissue disorders	1.52	0.90	2.57	2.07	1.98
Blood hematopoietic diseases	0.94	0.60	1.45	1.16	1.00
Congenital anomalies	0.81	0.46	1.32	0.96	0.75
Sensory diseases	0.00	0.00	0.00	0.01	0.07
Oral diseases	0.00	0.00	0.00	0.02	0.07
Injuries					
Accidents	31.38	19.21	43.98	32.99	18.54
Traffic accidents	9.96	5.94	14.78	10.64	6.48
Accidental falls	8.90	4.72	14.72	11.16	9.03
Accidental poisoning	1.47	0.70	2.71	2.17	2.45
Fire	0.29	0.00	0.63	0.43	0.53
Unintentional injuries	4.40	2.60	7.09	5.67	5.37
Suicide and sequelae	4.09	2.45	6.58	5.40	5.30
Homicide and sequelae	0.16	0.00	0.39	0.27	0.35
